# Efficacy of Knee Taping in addition to a Supervised Exercise Protocol to Manage Pain and Functional Status in Individuals with Patellofemoral Osteoarthritis: A Randomized, Controlled Clinical Trial

**DOI:** 10.1155/2022/2856457

**Published:** 2022-03-25

**Authors:** Mudasir Nazar Shah, Mohammad Abu Shaphe, Mohammed Qasheesh, Mohammad Kashif Reza, Ahmad H. Alghadir, Amir Iqbal, Priyadarshani Bhat

**Affiliations:** ^1^Department of Physical Therapy, College of Applied Medical Sciences, Jazan University, Jazan, Saudi Arabia; ^2^Department of Physiotherapy, Al-Karim University, Katihar, Bihar, India; ^3^Rehabilitation Research Chair, College of Applied Medical Sciences, King Saud University, Riyadh, Saudi Arabia; ^4^Department of Physiotherapy, Prakash Institute of Physiotherapy Rehabilitation and Allied Medical Sciences, Chaudhary Charan Singh University, Meerut, Uttar Pradesh, India

## Abstract

**Purpose:**

This study aimed to investigate the effect of knee taping in addition to a supervised exercise protocol on the pain intensity and functional status of individuals with patellofemoral osteoarthritis (PF OA).

**Methods:**

The study was based on a randomized, controlled pretest-posttest experimental group design. Following an initial screening, forty people with PF OA (mean age 55, range 40–60) were randomly assigned to one of two groups, Group A or Group B (*n* = 20 each). Group A underwent knee taping and participated in a supervised exercise program, whereas Group B only participated in a supervised exercise program. For four weeks, both groups received their prescribed treatment five consecutive days each week. At baseline (day 1 preintervention) and 4 weeks postintervention, the visual analog scale (VAS) and the Western Ontario and McMaster Universities Osteoarthritis Index (WOMAC) scores were obtained. To compare the effect of stipulated interventions within and between groups, paired and unpaired *t* tests were performed, with the level of significance set at *p* < 0.05.

**Results:**

When comparing the outcome scores at 4 weeks postintervention with baseline scores, the within-group analysis revealed significant mean differences for the outcomes within groups A (VAS: MD = −3.08–0.76; *T* = 9.70; *p* < 0.05 and WOMAC: MD = −7.05–0.81; *T* = 11.11; *p* < 0.05) and B (WOMAC: MD = −1.6–0.17; *T* = 2.35; *p* < 0.05), but a nonsignificant mean difference for the outcomes of VAS within group B (∆MD = 0.08 ± 0.03; *T* = −0.56; *p* > 0.05). Similarly, when the score of VAS (MD = −2.73–1.29; *T* = −9.17; *p* < 0.05) and WOMAC (MD = −5.95–1.63; *T* = −5.86; *p* < 0.05) were compared at 4 weeks postintervention, there was a significant mean difference between groups A and B.

**Conclusions:**

In people with patellofemoral osteoarthritis, combining knee taping with a supervised exercise protocol was more effective than the supervised exercise protocol alone in relieving pain and enhancing functional status.

## 1. Introduction

Osteoarthritis (OA) is a progressive disease that most often affects the large weight-bearing joints of the lower limbs, such as the knee and hip joints, and is recognized a global burden and a major health issue [1–3]. The overall prevalence (35%) of the knee OA, diagnosed by knee X-rays, increases with age [4]. The patellofemoral (PF) joint is particularly affected earlier than the tibiofemoral (TF) joint, becoming a major source of symptoms associated with symptomatic knee OA. Osteoarthritis is the result of multifactorial joint failure rather than a single disease [2]. In OA, the TF or PF joints are usually involved [2]. OA is a condition where the proliferative response acts after the degeneration of connective tissues and articular cartilage, which ends with the remodeling of the joint contour [5]. Multiple patterns of risk factors, including anatomical/structural, pathomechanical, physiological, and clinical characteristics, have been identified by various studies for the development of OA of the PF and TF joints [2, 5–7].

Evidence suggests that physical activities that exert more pressure on the PF joint, such as stairs ambulation, running, squatting, prolonged sitting, kneeling, quadriceps weakness, and patellar malalignment (patella alta/baja), produce symptoms like difficulty/pain on descending stairs, anterior-knee pain, and crepitus, and are more often linked with the development of PF OA rather than TF OA [7–10]. The quadriceps femoris muscle group's fibers cross through the patella at the PF joint to increase its effort-arm, enhancing the mechanical advantage of this muscle. As a result, the quadriceps femoris muscle controls and maintains patellar orientation in the trochlear groove of the femur to generate maximal power [9, 10]. Altered tracking of the patella during knee movement exerts unbalanced stresses on the patellar articular surface and causes the degeneration of its articular cartilage, which results in the development of PF OA [8, 9]. Therefore, tailored interventions addressing risk factors are required to decrease the progression and incidence of this disease.

The aim of conservative management in PF joint osteoarthritis is to minimize pain, limit functional impairments, and improve physical function in patients [10]. Cost-effective interventions with minimal side effects are encouraged [11]. Therefore, previous studies recommended using therapeutic taping techniques/interventions as a practical solution in PF joint OA [11, 12]. Knee taping has been proven to be a short-term intervention for knee pain in cases of PF OA and TF OA by correcting the misaligned PF joint and reducing the load over the inflamed soft tissue [11]. A cohort study revealed that patellar taping on the medial side for 4 days significantly reduced pain by 25% [12]. Most often, the pain and physical impairments/disability linked with the PF joint OA are due to the quadriceps weakness has been also benefited with the application of taping [11, 12].

Knee taping techniques aim to alleviate pain and enhance functional performance in participants with PF joint OA. Therapeutic exercises minimize the pain severity and enhance functional performance in participants with PF joint OA by aiming to enhance muscle strength, stabilize the joints, and maximize the range of motion (ROM) [12]. Quadriceps muscle weakness, stiffness in the PF joints, and decreased ROM in the joint, all of which add to pain and functional disability, are often observed in participants with PF joint OA. Therefore, overcoming these impairments is thought to minimize pain and enhance functional performance with PF joint OA [10–12].

To the best of our knowledge, the effectiveness of taping techniques or other therapeutic exercise protocols has been proven to be good in the management of PF OA [10–12]. However, the effect of taping techniques combined with supervised exercise protocols remains unknown. As a result, the purpose of this study was to investigate the effect of knee taping in combination with a supervised exercise protocol on pain intensity and functional status in individuals with PF OA. The research question of this study was as follows: “Does knee taping have an add-on effect to a supervised exercise protocol or is it equally as effective as a supervised exercise protocol alone in alleviating pain intensity and enhancing functional status in individuals with PF OA?”

## 2. Materials and Methods

### 2.1. Study Design

The study was based on a randomized, controlled pretest-posttest experimental group design.

### 2.2. Ethical Considerations

The study received an ethical clearance from the ethics subcommittee of our university (RRC-2019-16) and trial registration was obtained from a reputed trial agency (ClinicalTrials.gov PRS; ID: NCT04589871). The study followed the ethical standards for human research in accordance with the declaration of Helsinki. All the individuals signed and returned written informed consent.

### 2.3. Sample Size

A systematic random sampling technique was used in the study to obtain the sample. The VAS outcome score was used to estimate the effective sample size using computer software (*G*^*∗*^Power 3.1.9.4) based on the pilot study. It applied the a priori, *t* test for matched pairs to declare an effective sample size of 38 was needed in order to achieve a statistical power of 95% (0.95), with keeping the observed effect size 0.60, level of significance 0.05, mean differences 1.57, and standard deviation 2.61.

### 2.4. Selection of Participants

**Figure 1:**
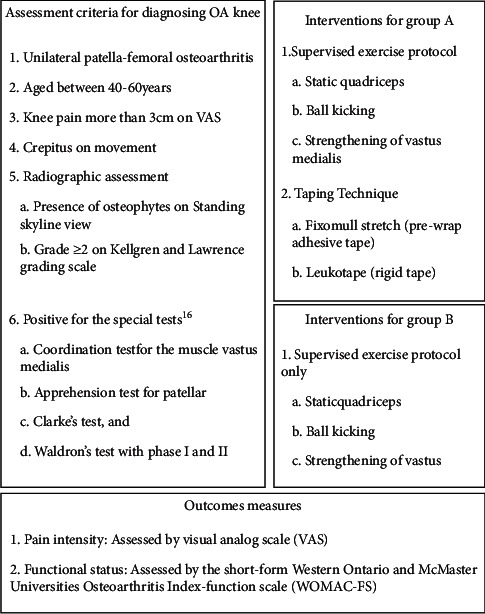
A brief explanation of the assessment, diagnostic criteria, interventions, and outcomes of the study.

Forty individuals diagnosed with PF OA as per the criteria indicated by the American College of Rheumatology (ACR) [13] were registered for this study from the outpatients' department at the rehabilitation center of King Saud University. Individuals diagnosed with unilateral PF OA varied in age from 40 to 60 years old, with a pain score greater than 3 cm on the visual analog scale, had crepitus when moving, and showed osteophytes on a skyline Laurin view (i.e., on standing with knee flexed at 45°) radiograph [14] (Kellgren/Lawrence grade ≥2) [15]; and positive results in specialized tests [16] (refer Figure 1) were included in this study. Individuals with TF joint OA; had patella alta/baja, rheumatoid arthritis/traumatic knee, and knee surgeries within six months; taking steroid injection; confirmed neurological deficiency, fragile skin over the knee, and tape allergy; and showed poor compliance were excluded from the study. All individuals were equally distributed into either group through a randomization process using the online tool named Randomization.com https://www.randomization.com/. The participants' group allocation was kept secret in a concealed envelop coded with a unique identifier number for each participant. The baseline scores for all the variables were taken by an assistant physiotherapist who was blinded to the study details and participant characteristics.

### 2.5. Outcome Measures

The outcomes (dependent variables) of this study, including the pain intensity and functional status of individuals with PF joint OA, were assessed by the visual analog scale (VAS) and the reduced Western Ontario and McMaster Universities Osteoarthritis Index function scale (reduced WOMAC-FS), respectively. The visual analog scale is a reliable, validated, responsive, and frequently used subjective measure for the level of pain. The participant was given a 10 cm horizontal line with the numbers 0 (no pain) and 10 (worst pain) on either end to describe his or her level of discomfort by drawing a vertical line between the two ends [17]. The test-retest reliability (ICC, 95% CI), standard error of measurements (SEM), and minimal detectable changes (MDC) were 0.97, 0.03, and 0.08, respectively [18, 19].

The short-form WOMAC-FS is also a reliable, valid, responsive alternative to traditional WOMAC and an often-used patient-reported subjective functional health measure for patients with hip and knee OA [19–22]. It is available in 5-point Likert, 11-point numerical rating, and 100 mm visual analogue scale (VAS) formats and comprises 7-items in functional dimensions [21–23]. The short-form WOMAC-FS has shown excellent test-retest reliability (Lin's concordance correlation coefficient (*ρ*c): 0.85 to 0.94), good internal consistency (Cronbach alpha: 0.88 to 0.95) and a high correlation (Spearman's correlation coefficient *r* = 0.96) with the original WOMAC [19, 21–24].

### 2.6. Procedures

**Figure 2:**
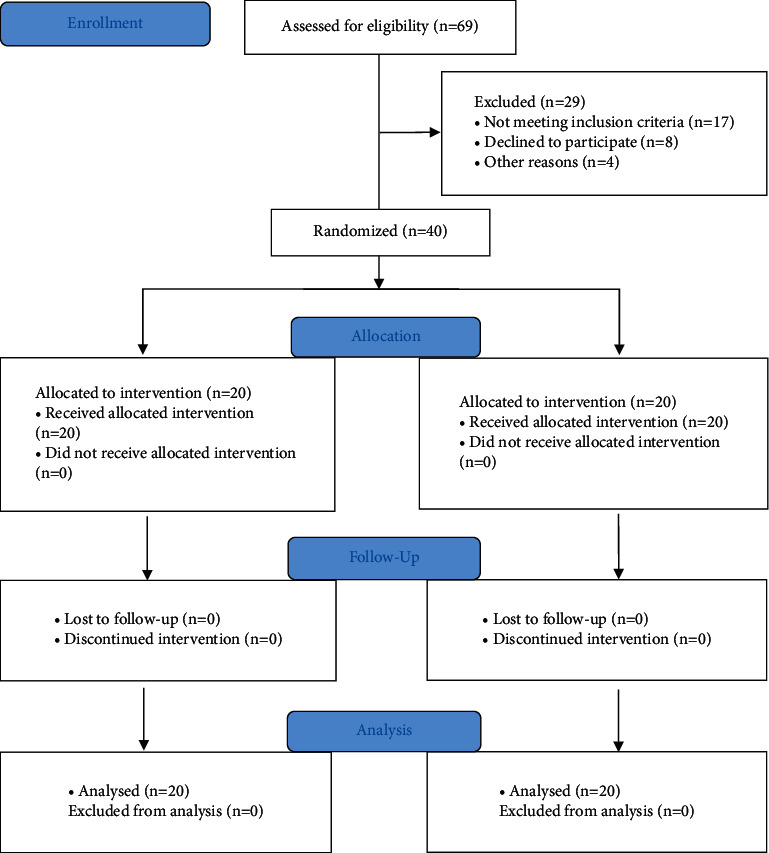
A CONSORT flow diagram depicting enrollment, group allocation, follow-up, and analysis of the study procedures.

A total of 40 individuals with unilateral PF OA were screened and recruited in the study based on inclusion and exclusion criteria. A written informed consent was obtained from all the individuals before the commence of the study. After taking the demographic details and baseline measurements for all the outcome variables, all individuals were randomly divided into experimental (Group A) and control groups (Group B). Knee taping was applied in Group A in addition to a supervised exercise program, while Group B simply got a supervised exercise program. For four weeks, both groups got the intervention five days a week. At 4 weeks' postintervention, the same assessor collected the outcome data for all the individuals participating in the study. The procedures of the study, including enrollment, allocation, follow-up, and analysis, are presented in a CONSORT (2010) flow diagram in Figure 2.

Additionally, Figure 1 provides a brief explanation of the assessment, diagnostic criteria, interventions, and outcomes of the study.

### 2.7. Interventions

#### 2.7.1. Supervised Exercise Protocol

*(1) Static Quadriceps Exercise*. The exercise was performed at the terminal range of knee extension (0–15°) while compressing the ball with hip adduction (10 min). The participant was asked to tighten the thigh muscles, pull the kneecap up, hold for a count of 10, and then let go. An isometric contraction of quadriceps by the participant guiding the patellar tracking proximally; kept holding for a count of 10; and then let go. A single set consists of 18 repetitions with a 30 second rest period between two consecutive repetitions.

*(2) Ball Kicking Exercise*. Patients performed ball kicking against the wall for 5 min. They were instructed to rest for 30 sec after every minute and completed 4 sets of 12 repetitions with a 30 sec rest period between two consecutive sets.

*(3) Vastus Medialis Strengthening Exercise*. Patients were asked to perform hip adduction and internal rotation with knee extension in a sitting position on a straight back chair for 1 minute (10 minutes). One set of 10 repetitions included resistance exercises for the quadriceps muscles by placing a weight cuff around the participant's ankle and using a quadriceps table.

*(4) Manual Resistance Exercise*. The participant was instructed to sit on a quadriceps table and asked to extend the knee against the resistance offered by the therapist. One set of 10 repetitions was performed.

*(5) Weight Cuff Exercise*. The participant was made to sit on a quadriceps table and asked to extend the knee against the resistance provided by the cuff weight, completing 1 set of 10 repetitions.

*(6) Quadriceps Table Exercise*. The participant was made to sit on a quadriceps table and asked to extend the knee against the resistance offered by the quadriceps table will do 1 set of 10 repetitions.

*(7) Instruction*. The participant was asked to inform the therapist about any pain or discomfort experienced during the exercise.

The total time for the supervised exercise protocol was 30–35 minutes.

#### 2.7.2. Taping Technique [11]

Taping was done using Fixomull stretch and leukotape (rigid tape) to guide the patellar trajectory alignment during the motions. Individuals were instructed to lie down in a supine position with their knees bent slightly. To protect the skin and give some checking force, Fixomull stretch was utilized as an adhesive prewrap. Strips of stiff tape served as the main check strap (leukotape). Starting at the outside edge of the kneecap and pulling inside, the tape was applied to the kneecap, ending at the rear of the inside of the knee. Distal to the patella, two further pieces of tape (Fixomull stretch and leukotape) were placed to empty the infrapatellar fat pad.

McConnell suggested 2 components of patellar orientation: the glide component and the tilt component. The therapist aligned the gliding component by securely taping the lateral border of the patella to the medial femoral condyle and the tilt component by taping the center of the patella to the medial femoral condyle [11].

### 2.8. Statistical Analysis

A statistical package for windows version 21 (SPSS, IBM Inc., Chicago, IL) was used for the data analysis for the variables. The level of pain and functional status was assessed by using the VAS and a reduced WOMAC functional scale at preintervention (baseline) and 4 weeks postintervention for the individuals of both groups A and B. Descriptive analysis (mean, standard deviation) was used to describe the demographic characteristics. Paired and unpaired *t* tests were used to evaluate the statistically significant differences in the outcome scores within group and between groups, respectively. For all the tests, 0.05 (*p* value) was set as the level of significance.

## 3. Results

Sixty-nine participants (41 males and 28 females) with PF OA were screened for the study. Out of 69 participants, 29 were excluded from the study: 17 did not meet the inclusion criteria, 8 declined to participate for genuine reasons, and 4 declined to participate without reason. A total of 40 participants (27 males and 13 females) were included in this study. Descriptive details for the demographic characteristics and baseline VAS and WOMAC functional status scores are described in Table 1.

### 3.1. Within-Group Analysis

Within-group comparisons indicated statistically significant mean differences in outcomes within Groups A (VAS: ∆MD = −3.08 ± −0.76; *T* = 9.70; *p* < 0.05 and WOMAC functional status: ∆MD = −7.05 ± −0.81; *T* = 11.11; *p* < 0.05) and B (WOMAC functional status: ∆MD = −1.6 ± −0.17; *T* = 2.35; *p* < 0.05); however, a nonsignificant mean difference was discovered within Group B for the outcome of VAS (∆MD = 0.08 ± 0.03; *T* = −0.56; *p* > 0.05) scores when comparing the outcome scores at 4 weeks postintervention with baseline scores, as described in Table 2.

### 3.2. Between-Groups Analysis

Similarly, a significant mean difference was found for the VAS (∆MD = −2.73 ± −1.29; *T* = −9.17; *p* < 0.05) and WOMAC functional status (∆MD = −5.95 ± −1.63; *T* = −5.86; *p* < 0.05) scores when compared between Groups A and B at 4 weeks postintervention, as shown in Table 3.

## 4. Discussion

This study was done to evaluate the level of pain and functional status in patients with PF OA after delivering a strengthening exercise with taping technique to group A and strengthening exercise only to group B and to see which was more beneficial. After including these techniques to resolve the condition, the required treatment time was increased to speed up the recovery rate by reducing the required session. There is also definite benefit of greater satisfaction on patients' side due to hands on, caring by the therapist. As a result, it is sufficient to consider this approach as an important component of the PF OA treatment plan. However, the quadriceps femoris muscle fibers pass over the patella allows the PF joint's lever-arm to be increased and the mechanical advantage to be maximized. Hence, the quadriceps femoris muscle is able to effectively control and maintain patellar movement in the trochlear groove of the femur to generate maximal power [9, 10]. In contrast, altered tracking of the patella during knee movement exerts uneven stresses on the patellar articular surface, causing the degeneration of its articular cartilage, which results in the development of PF joint OA [8, 9, 25].

The purpose of this study was to investigate the efficacy of knee taping when used in conjunction with a supervised exercise protocol to treat PF OA. The experimental group (Group A) received both taping and strengthening exercise, while the control group (Group B) received only strengthening exercise. The treatment for each group was given for 4-weeks.

The findings of the study reveal that each group improved significantly in terms of pain and functional status, except for the outcome VAS in Group B. Moreover, Group A demonstrated higher pain relief and functional status compared to Group B. This finding demonstrates that the quadriceps femoris muscle gained control over patellar tracking with the use of a taping technique that aided to patella maintaining proper tracking in the trochlear groove of the femur. Gabriel YF Ng and Jenny MF Cheng revealed that the taping of patella significantly reduced the PF pain however, lessen the activities of VMO and VL fibers in the quadriceps muscle group, which is opposite to the findings of the current study [25]. In another study, Noako Aminaka et al. evaluated the effectiveness of knee taping on patellar pain and neuromuscular control among individuals with patellofemoral pain syndrome (PFPS) and concluded that patellar taping significantly alleviates PF pain and enhances VMO activity [26]. Another group of researchers assessed the effects of several physical therapies on pain in patients with PF OA and discovered that combining patellar taping with biofeedback was useful and successful in lowering PF joint pain in the short-term [27].

Quadriceps and VMO strengthening exercises result in tendon strengthening by stimulating mechanoreceptors in tenocytes to make collagen and regulate the large proportion of glycosaminoglycan, which may enhance tendon alignment and increase collagen formation. Cross-linkage formation facilitates improvements in tensile strength. Posttraumatic accumulation of blood in a damaged area stimulates neovascularization for the purpose of revascularization and long-term healing [25, 26]. In addition, stretching exercises are effective through the lengthening of the musculotendon unit, which subsequently lessens the strain during joint motion. However, strengthening exercises are effective through loading within the muscle tendon, resulting in increased tensile strength and muscle hypertrophy [25–27].

In a previous study, researchers included thirty patients experiencing PFPS and divided them randomly into three groups. They concluded in their report that combining patellar exercise with a standardized exercise program for four weeks was more efficient in decreasing pain and function in individuals with PFPS than a standardized exercise program alone [28]. Another study enrolled 81 individuals with anterior PFPS and randomly allocated them to one of four groups. The discharge rate, the visual analog scale (VAS) rating for pain, the WOMAC score for functional output, the Hospital Anxiety and Depression Scale score, and quadriceps strength were all used to determine patient satisfaction. The quadriceps strength significantly increased in the group that received exercise, taping, and education and was superior to the group that received taping alone among all the participants with PF OA over 3-month postintervention. From the above study, we can conclude that exercise and taping should be given in a single group to enhance the effect, and thus the above studies are supporting the result of the current study [29]. In contrast, Kowall et al. conveyed in their reports that the patellar taping as an add-on to the standardized physiotherapy program added no improvement in pain intensity, quadricep muscle isokinetic strength, and EMG activity when compared with the standardized physiotherapy program alone [30].

The results of the experimental group of the current study were supported by various studies [30, 31]. According to the findings of these numerous trials, individually, patellar taping and a standardized exercise program were beneficial in minimizing pain intensity and enhancing functional status in individuals with PF joint OA. However, it was shown that combining patellar taping and a structured exercise program was more successful than either program alone. This effect was hypothesized because taping techniques enhanced patellar navigation inside the femoral groove, soft tissue stretching around the lateral knee, and VMO strengthening of the medial knee [30, 31]. The McConnell patellar taping regimen's true objective is to direct patellar tracking toward the medial side of the femoral groove, allowing for pain-free mobility during an exercise program [11].

Although both the experimental and control groups experienced significant decline in pain intensity and gain in functional status, the experimental group outperformed the control group in terms of decreased pain intensity and enhanced functional status in individuals with PF OA. Hence, patellar taping technique and standardized exercise plan can successfully provide pain relief to the individuals with PF OA.

This study has a significant and consistent finding: the application of the taping technique and supervised exercise in the management of PF joint OA led to an improvement in pain relief and functional status. Although the verification of MC Connell's postulated mechanism for reducing pain is as elusive as the cause of PF joint pain, the favorable effects of the taping technique justify its continued usage in the clinic. The clinical significance of pain reduction also impacts the exercise arena. Pain, as McConnell points out, has an inhibitory influence on the quality of muscular contraction and is known to be a major contributor to functional limitations. Patellar taping reduces patellofemoral discomfort, allowing more improvements in functional status to be obtained. Patellar taping reduces PF joint pain by correcting patellar trajectory through the femoral groove, allowing more improvements in functional status to be obtained. Additionally, the results of this study indicated that the taping technique and supervised exercise protocol used in the experimental group (Group A) led to a significant improvement in the participant's pain relief and functional status; thus, it can be concluded that knee taping and supervised exercise can be used effectively to treat individuals with PF OA to alleviate pain and improve functional status.

### 4.1. Limitation

This study was done during a brief period of time, and a longer-term investigation may be required to confirm the findings. A follow-up study could be performed to determine the probability of recurrence of the condition. A follow-up study could also ensure the long-term effectiveness of the treatment given. The sample size could be increased to further establish the efficacy of the treatment. The effectiveness of this technique is highly dependent on the proficiency and experience of the therapist.

## 5. Conclusions

The study revealed that combining knee taping with supervised exercise is more efficacious than supervised exercise alone in terms of lowering pain intensity and enhancing functional status in individuals with PF OA. Thus, physiotherapists may utilize knee taping techniques in conjunction with supervised exercise protocols to alleviate pain and enhance functional status in individuals with PF OA.

### 5.1. Future Research

Future study is critical, with a larger sample size and proper follow-up. Additionally, it is proposed to evaluate the effectiveness of knee/patellar taping on the quadriceps with and without EMG/NMES activity. Furthermore, the role of complementary alternative medicines in acute and chronic diseases (such as inflammatory, degenerative, hypertensive, and diabetic conditions, etc.) is very important and has been proven to be effective in pain relief, improving muscle functions/performances (i.e., strength and endurance), wellbeing, and gaining popularity in recent years [32–36]. However, its usage in physiotherapy settings has been rare and limited due to a lack of exploration in this direction. Therefore, the physical therapist should utilize a combination approach of physiotherapeutic intervention with complementary alternative medicines that might be getting more productive results in reducing knee pain and improving muscular performance in various conditions.

## Figures and Tables

**Table 1:** Demographic characteristics and baseline scores of all variables for all the participants (*N* = 40).

#	Variables (*N* = 40)	Group A (mean ± SD)	Group B (mean ± SD)	*P* value
1	Age (years)	55.55 ± 3.80	55.3 ± 3.88	1.00
2	Gender			
	Male	13 (65%)	14 (70%)	n/a
	Female	7 (35%)	6 (30%)	n/a
3	BMI (kg/m^2^)	24.79	24.19	1.00
4	X-ray			
	Osteophytes	Present	Present	n/a
	K & L gradeﬞ	2-3	2-3	n/a
5	COM	Yes	Yes	n/a
6	VAS	6.00 ± 1.73	5.57 ± 2.23	1.00
7	WOMAC functional status	20.95 ± 3.88	21.45 ± 4.87	1.00

SD: standard deviation; VAS: Visual Analog Scale; WOMAC: The Western Ontario and McMaster Universities Osteoarthritic Index; K/L: Kellgren and Lawrence grading of knee OA; COM: crepitus on movement; #: serial numbers.

**Table 2:** Comparison of VAS and WOMAC functional status (mean ± SD) scores within Groups A and B using paired *t*-test.

				Paired *t*-test	
Outcomes	Baseline	4 weeks	∆MD	*t* value	*p* value
*Group A (N* *=* *20)*					

VAS	6.00 ± 1.73	2.92 ± 0.97	−3.08 ± −0.76	9.70	<0.05^*∗*^
WOMAC-FS	20.95 ± 3.88	13.9 ± 3.07	−7.05 ± −0.81	11.11	<0.05^*∗*^

*Group B (N* *=* *20)*					

VAS	5.57 ± 2.23	5.65 ± 2.26	0.08 ± 0.03	−0.56	>0.05
WOMAC-FS	21.45 ± 4.87	19.85 ± 4.70	−1.6 ± −0.17	2.35	<0.05^*∗*^

WOMAC-FS: The Western Ontario and McMaster Universities Osteoarthritic Index—Functional Status; ∆MD: mean difference; SD: standard deviation; ^*∗*^significant value if *p* < 0.05.

**Table 3:** Comparison of VAS and WOMAC functional status (mean ± SD) scores between Groups A and B using unpaired *t*-test.

Outcomes	Scores at 4 weeks postintervention			Unpaired *t*-test	
	Group A (*n* = 20)	Group B (*n* = 20)	∆MD (A vs. B)	*t* value	*p* value
VAS	2.92 ± 0.97	5.65 ± 2.26	−2.73 ± −1.29	−9.17	<0.05^*∗*^
WOMAC-FS	13.9 ± 3.07	19.85 ± 4.70	−5.95 ± −1.63	−5.86	<0.05^*∗*^

^*∗*^Significant value if *p* < 0.05; ∆MD: mean difference; SD: standard deviation; WOMAC-FS: The Western Ontario and McMaster Universities Osteoarthritic Index—functional status.

## Data Availability

The dataset supporting the conclusions of this article is available through the corresponding author on reasonable request.

## References

[1] Cross M., Smith E., Hoy D. (2014). The global burden of hip and knee osteoarthritis: estimates from the global burden of disease 2010 study. *Annals of the Rheumatic Diseases*.

[2] Stefanik J. J., Guermazi A., Roemer F. W. (2016). Changes in patellofemoral and tibiofemoral joint cartilage damage and bone marrow lesions over 7 years: the Multicenter Osteoarthritis Study. *Osteoarthritis and Cartilage*.

[3] Kobayashi S., Pappas E., Fransen M., Refshauge K., Simic M. (2016). The prevalence of patellofemoral osteoarthritis: a systematic review and meta-analysis. *Osteoarthritis and Cartilage*.

[4] Pereira D., Peleteiro B., Araújo J., Branco J., Santos R. A., Ramos E. (2011). The effect of osteoarthritis definition on prevalence and incidence estimates: a systematic review. *Osteoarthritis and Cartilage*.

[5] Duncan R., Peat G., Thomas E., Wood L., Hay E., Croft P. (2009). Does isolated patellofemoral osteoarthritis matter?. *Osteoarthritis and Cartilage*.

[6] Hinman R. S., Crossley K. M. (2007). Patellofemoral joint osteoarthritis: an important subgroup of knee osteoarthritis. *Rheumatology*.

[7] Mills K., Hunter D. J. (2014). Patellofemoral joint osteoarthritis: an individualised pathomechanical approach to management. *Best Practice & Research Clinical Rheumatology*.

[8] Peat G., Duncan R. C., Wood L. R., Thomas E., Muller S. (2012). Clinical features of symptomatic patellofemoral joint osteoarthritis. *Arthritis Research & Therapy*.

[9] Schiphof D., van Middelkoop M., de Klerk B. M. (2014). Crepitus is a first indication of patellofemoral osteoarthritis (and not of tibiofemoral osteoarthritis). *Osteoarthritis and Cartilage*.

[10] Bolgla L. A., Boling M. C. (2011). An update for the conservative management of patellofemoral pain syndrome: a systematic review of the literature from 2000 to 2010. *International Journal of Sports Physical Therapy*.

[11] Hinman R. S., Crossley K. M., McConnell J., Bennell K. L. (2003). Efficacy of knee tape in the management of osteoarthritis of the knee: blinded randomised controlled trial. *BMJ*.

[12] Collins N. J., Barton C. J., Van Middelkoop M. (2018). 2018 Consensus statement on exercise therapy and physical interventions (orthoses, taping and manual therapy) to treat patellofemoral pain: recommendations from the 5th International Patellofemoral Pain Research Retreat, Gold Coast, Australia, 2017. *British Journal of Sports Medicine*.

[13] Altman R., Asch E., Bloch D. (1986). Development of criteria for the classification and reporting of osteoarthritis: classification of osteoarthritis of the knee. *Arthritis & Rheumatism*.

[14] Bucklandwright C. (2006). Which radiographic techniques should we use for research and clinical practice?. *Best Practice & Research Clinical Rheumatology*.

[15] Kellgren J. H., Lawrence J. S. (1957). Radiological assessment of osteo-arthrosis. *Annals of the Rheumatic Diseases*.

[16] Nijs J., Van Geel C., Van der auwera C., Van de Velde B. (2006). Diagnostic value of five clinical tests in patellofemoral pain syndrome. *Manual Therapy*.

[17] Delgado D. A., Lambert B. S., Boutris N. (2018). Validation of digital visual analog scale pain scoring with a traditional paper-based visual analog scale in adults. *Journal of the American Academy of Orthopaedic Surgeons. Global Research &Reviews*.

[18] Alghadir A., Anwer S., Iqbal A., Iqbal Z. (2018). Test–retest reliability, validity, and minimum detectable change of visual analog, numerical rating, and verbal rating scales for measurement of osteoarthritic knee pain. *Journal of Pain Research*.

[19] Alghadir A., Anwer S., Iqbal Z. A., Alsanawi H. A. (2016). Cross-cultural adaptation, reliability and validity of the Arabic version of the reduced Western Ontario and McMaster Universities Osteoarthritis index in patients with knee osteoarthritis. *Disability & Rehabilitation*.

[20] Woolacott N. F., Corbett M. S., Rice S. J. C. (2012). The use and reporting of WOMAC in the assessment of the benefit of physical therapies for the pain of osteoarthritis of the knee: findings from a systematic review of clinical trials. *Rheumatology*.

[21] Whitehouse S. L., Lingard E. A., Katz J. N., Learmonth I. D. (2003). Development and testing of a reduced WOMAC function scale. *Journal of Bone & Joint Surgery, British Volume*.

[22] Yang K. G. A., Raijmakers N. J. H., Verbout A. J., Dhert W. J. A., Saris D. B. F. (2007). Validation of the short-form WOMAC function scale for the evaluation of osteoarthritis of the knee. *Journal of Bone & Joint Surgery, British Volume*.

[23] Gorial F. I., Sabah S. A.-S. A., Kadhim M. B., Jamal N. B. (2018). Functional status in knee osteoarthritis and its relation to demographic and clinical features. *Mediterranean Journal of Rheumatology*.

[24] Escobar A., Vrotsou K., Bilbao A., Quintana J. M. A., García Pérez L., Herrera-Espiñeira C. (2011). Validación de una escala reducida de capacidad funcional del cuestionario WOMAC. *Gaceta Sanitaria*.

[25] Ng G. Y., Cheng J. M. (2002). The effects of patellar taping on pain and neuromuscular performance in subjects with patellofemoral pain syndrome. *Clinical Rehabilitation*.

[26] Aminaka N., Gribble P. A. (2005). A systematic review of the effects of therapeutic taping on patellofemoral pain syndrome. *Journal of Athletic Training*.

[27] Grabowski S. R., Tortora G. J. (2008). *Principles of Anatomy and Physiology*.

[28] Whittingham M., Palmer S., Macmillan F. (2004). Effects of taping on pain and function in patellofemoral pain syndrome: a randomized controlled trial. *Journal of Orthopaedic & Sports Physical Therapy*.

[29] Clark D. I., Downing N., Mitchell J., Coulson L., Syzpryt E. P., Doherty M. (2000). Physiotherapy for anterior knee pain: a randomised controlled trial. *Annals of the Rheumatic Diseases*.

[30] Kowall M. G., Kolk G., Nuber G. W., Cassisi J. E., stern S. H. (1996). Patellar taping in the treatment of patellofemoral pain. *The American Journal of Sports Medicine*.

[31] Quilty B., Tucker M., Campell R., Dicppe P. (2003). Quadriceps exercises and patellar taping for knee osteoarthritis with predominant patello femoral joint involvement randomized controlled trial. *Journal of Rheumatology*.

[32] Su Y., Yao S., Zhou Z. J., Wu C., Wang I. L., Lai C. Y. (2022). Effect of acupuncture on time-dependent of muscle endurance in female elbow joint: a randomized controlled trial. *Evidence-based Complementary and Alternative Medicine*.

[33] Seyed Hashemi M., Hashempur M. H., Lotfi M. H. (2019). The efficacy of asafoetida (Ferula assa-foetida oleo-gum resin) versus chlorhexidine gluconate mouthwash on dental plaque and gingivitis: a randomized double-blind controlled trial. *European Journal of Integrative Medicine*.

[34] Dong X., Shi Z., Ding M., Yi X. (2021). The effects of qigong for hypertension: a meta-analysis of randomized controlled trials. *Evidence-based Complementary and Alternative Medicine*.

[35] Nayebi N., Esteghamati A., Meysamie A. (2019). The effects of a Melissa officinalis L. based product on metabolic parameters in patients with type 2 diabetes mellitus: a randomized double-blinded controlled clinical trial. *Journal of Complementary & Integrative Medicine*.

[36] Ilkhani R., Bigdeli M., Mohkam M. (2021). Topical use of saussurea costus (falc.) lipsch. (qost) oil in pediatric nocturnal enuresis in comparison with sesame oil, a randomized double-blind clinical trial. *Traditional and Integrative Medicine*.

